# Protective and Therapeutic Effects of Lactic Acid Bacteria against Aflatoxin B1 Toxicity to Rat Organs

**DOI:** 10.3390/microorganisms11071703

**Published:** 2023-06-29

**Authors:** Hayat Ashi, Meshal H. K. Almalki, Enas A. Hamed, Wafaa S. Ramadan, Tahani F. H. Alahmadi, Outour Tariq Alami, Sara H. Arafa, Atheer K. Alshareef, Fatimah S. Alsulami, Areej F. Alharbi, Manahil S. Al-Harbi, Ebtehal H. Alqurashi, Shirin Aashi, Youssef A. Alzahrani, Khaled Elbanna, Hussein H. Abulreesh

**Affiliations:** 1 Department of Biology, Faculty of Applied Science, Umm Al-Qura University, Makkah 21955, Saudi Arabia; dr.ht.111@hotmail.com (H.A.); mhmaleki@uqu.edu.sa (M.H.K.A.); tootaa.2030@gmail.com (T.F.H.A.); outour-alami@hotmail.com (O.T.A.); arafa.sa@hotmail.com (S.H.A.); alal-2021@hotmail.com (A.K.A.); fatima.alsulami@hotmail.com (F.S.A.); areej.f.alharbi@hotmail.com (A.F.A.); s44285749@st.uqu.edu.sa (M.S.A.-H.); ebtehal373@yahoo.com (E.H.A.); kab00@fayoum.edu.eg (K.E.); 2 Research Laboratories Unit, Faculty of Applied Science, Umm Al-Qura University, Makkah 21955, Saudi Arabia; 3 Department of Medical Physiology, Faculty of Medicine, Assiut University, Assiut 71515, Egypt; eah3a2010@aun.edu.eg; 4 Department of Anatomy, Faculty of Medicine, King Abdulaziz University, Jeddah 21589, Saudi Arabia; 5 College of Pharmacy, King Abdulaziz University, Jeddah 21589, Saudi Arabia; sheren_141@hotmail.com (S.A.); youssefalzahrani18@gmail.com (Y.A.A.); 6 Department of Agricultural Microbiology, Faculty of Agriculture, Fayoum University, Fayoum 63514, Egypt

**Keywords:** aflatoxin, lactic acid bacteria, liver, kidney, protective, rats, spleen, structure, testes, therapeutic

## Abstract

Background: Aflatoxin (AF), a metabolite of *Aspergillus flavus*, is injurious to vital body organs. The bacterial defense against such mycotoxins has attracted significant attention. Lactic acid bacteria (LAB) are known to ameliorate AF toxicity. Methods: Thirty adult male rats were divided into six groups (five each) to perform the experiments. The control (Co) group was fed a basal diet and water. Each of the following periods lasted 21 days: the milk (MK) group orally received milk (500 µL); LAB suspension (500 µL) containing 10^7^ cfu/mL was orally provided to the LAB group; AF (0.5 mg/kg) was orally given to the AF group; and a combination of AF and LAB was administered to the AF + LAB group. The AF/LAB group was initially given AF for 21 days, followed by LAB for the same period. Finally, the rats were dissected to retrieve blood and tissue samples for hematological, biochemical, and histological studies. Results: The results revealed a significant decrease in RBCs, lymphocytes, total proteins, eosinophil count, albumin, and uric acid, whereas the levels of WBCs, monocytes, neutrophils, creatinine, urea, aspartate aminotransferase, alkaline phosphatase, alanine aminotransferase, lactate dehydrogenase, and creatinine kinase significantly increased in the AF group in comparison to the control group. The histological examination of the AF group revealed necrosis and apoptosis of the kidney’s glomeruli and renal tubules, nuclei vacuolization and apoptosis of hepatocytes, congestion of the liver’s dilated portal vein, lymphoid depletion in the white pulp, localized hemorrhages, hemosiderin pigment deposition in the spleen, and vacuolization of seminiferous tubules with a complete loss of testis spermatogenic cells. Meanwhile, protective and therapeutic LAB administration in AF-treated rats improved the hematological, biochemical, and histological changes. Conclusions: The study revealed LAB-based amelioration to AFB1-induced disruptions of the kidney, liver, spleen, and testis by inhibiting tissue damage. The therapeutic effects of LAB were comparatively more pronounced than the protective effects.

## 1. Introduction

Toxigenic-fungi-induced mycotoxins in foods and feedstuffs could lead to significant impacts on human and animal health. Aflatoxins (AF) are secondary metabolites of *Aspergillus nominus*, *Aspergillus parasiticus*, and *Aspergillus flavus* that are naturally found in human food and animal feed. The large AF family mainly includes AFB_1_, AFB_2_, AFG_1_, and AFG_2_ mycotoxins [1]. There is a global presence of *Aspergillus* mycotoxins in cereals, milk, and animal feed. Aflatoxins have caused several aflatoxicosis outbreaks in humans and animals, which resulted in serious human health issues, increased veterinary care costs, and reduced livestock production [2]. Aflatoxin B_1_ (AFB_1_) has been implicated as an etiological factor for developing liver cancer and other chronic liver diseases [3]. AF immunotoxicity in various laboratory and domestic animals has been reported [4].

Probiotic-bacteria-based microbial food supplements are known to exert positive health and therapeutic effects such as an improved immune system, reduced lactose intolerance, decreased cancer risk, enhanced intestinal tract health, pathogen antagonization, and increased synthesis and bioavailability of nutrients [5]. A broad range of bacterial genera and species are considered probiotics. The consumption of lactic acid bacteria (LAB) (Bifidobacteria and lactobacilli) helps in cancer prevention and the regulation of immune response [6]. Probiotic LAB is an important component of gut flora. These Gram-positive bacteria are characterized as bile-tolerant, acid-resistant, catalase-lacking, non-respiring, and non-spore-forming rods or cocci. They produce various antimicrobials, including hydrogen peroxide, organic acids, and biologically active proteins (bacteriocins), which have a status of GRAS (generally recognized as safe) [7]. LAB is commonly used in yogurt preparation, and its fermented products modulate immunity in gut lymphoid cells to promote health. Bifidobacteria and LAB dairy strains are known to successfully bind AFB1 in a buffered solution [8]. Dairy products are food that commonly contains probiotic microorganisms. Jebali et al. [9] have reported LAB-based anti-immunotoxin/immunomodulatory effects in mycotoxin-treated mice. LAB increases *α*-esterase and bone marrow cells, which helps to counter mycotoxin’s effects. LAB treatment also enhances antibody titer circulation and plaque-forming cell levels to initiate the humoral immune response arm against mycotoxins [10].

This study elaborates on the protective and therapeutic impacts of LAB administration against aflatoxin B1 toxicity to various rat organs, including kidneys, liver, spleen, and testes. We explored the therapeutic and protective potential of a LAB strain, *Lactobacillus rhamnosus*, recovered from fermented camel milk products on rat organs that had been exposed to aflatoxin. This strain has been recovered from fermented dairy products of camel milk, which showed high antimicrobial potential against a wide range of foodborne pathogens; it also improved the mucosal immune response through an increase in the expression of TLR2 and IFNγ nRNA in mice intestine, as well as increasing the production of IgG, IgM, and IgA in mice blood sera.

## 2. Material and Methods

### 2.1. Aflatoxin

#### 2.1.1. Fungal Isolate

To retrieve a local isolate, a loopful of spores was scraped from moldy bread and cultured on potato dextrose agar (PDA). The PDA contained potato extract (4.0 g) (fresh, unpeeled potatoes), glucose (20 g) (Merck KGaA, Darmstadt, Germany), and agar (20 g) (Oxoid, Basingstoke, UK) in distilled water (1000 mL) [11]. The agar was dissolved by boiling the mixture, followed by sterilization before pouring it onto plates. Sabouraud dextrose agar (SDA) (HiMedia, Mumbai, India) was also used for the parallel culturing of the isolate. Both media were incubated for five to seven days at 25 ± 2 °C [12]. Then, a cork borer was used to inoculate a fungal disk on *Aspergillus* differentiation agar. This agar was prepared by adding 10 g of yeast extract (HiMedia, Thane, India), 15 g of tryptone (HiMedia), 0.5 g of ferric citrate (Merck KGaA), and 15 g of agar (Oxoid) to distilled water (1000 mL). The media were boiled and autoclaved, and the inoculated plates were incubated for five to seven days at 25 ± 2 °C [13].

#### 2.1.2. Maintaining and Storage of Isolate

*A. flavus* isolate was cultured on SDA plates and incubated for five to seven days at 25 ± 2 °C. Then, *A. flavus* colonies were inoculated into sterile glycerol solution (15%) (HiMedia) in sterile microfuge tubes (250 µL) followed by cryogenic storage (−80 °C) to maintain the vitality of fungal spores for longer periods [14].

#### 2.1.3. Production, Extraction, and Determination of Aflatoxin

*A. flavus* isolate was cultivated in SDB for 5–14 days under shaking and aerobic conditions at 25 ± 2 °C. Then, the culture was filtrated through sterile filter papers (MN 615—∅ 150 mm) using a sterile funnel. The filtrates were collected in sterile conical tubes (15 mL, Plastilab, Roumieh, Lebanon). Fungal cell walls were weakened by placing filtrates at cryogenic temperature (−80 °C) overnight. It facilitated solvent penetration into the cell to extract secondary products [15]. The contents were transferred to a hydrophilic bottle containing methanol (15 mL) (Merck KGaA) and subjected to ultrasonic vibration for half an hour, followed by shaking at 100 rpm in an orbital shaker [14]. This step was repeated multiple times, and the extract was filtered through a micropore filter paper-containing sterile glass funnel. The filtrate was evaporated under nitrogen flow to concentrate, which was dissolved in methanol (1.5 mL) and analyzed with HPLC (Shimadzu, Kyoto, Japan) and GC (Shimadzu) [16]. A standard crude aflatoxin B1 extract (Courtesy of Prof. Ahmed Abdelmalek, Moubasher Mycological Center, Assiut University, Assiut, Egypt) was used to compare the extracted toxin.

#### 2.1.4. Testing of Aflatoxin Production

Aflatoxin production was confirmed by following the ammonia vapor method, which turns the color of toxin-secreting colonies into pink on SDA/PDA culture plates. Ammonia solution (25%) (Merck KGaA) was prepared by adding ammonia (25 mL) to a flask containing distilled water (75 mL). An amount of 0.2 mL of this solution was placed on the Petri dish and incubated at 25 °C for 24 h, developing a red color at the bottom of fungal colonies [17]. 

#### 2.1.5. Gas Chromatography (GC) Analysis

Gas chromatography uses gas as the mobile phase, and liquid is confined to solid particles (stationary phase). During GC analysis, the polyphase (Diphenyl 35%/Dimethylsiloxane 65%) type robust and medium polarity stationary phase is required to withstand the impacts of heating and silylating agent. A fast-temperature program was applied for a good aflatoxin separation. Initial and final temperatures were adjusted at 50 and 250 °C, respectively, with a carrier gas flow rate of 2 mL/min and a heating rate of 15 °C/min. UV absorbance of aflatoxin was monitored at a wavelength of 272 nm. GC analysis was carried out in a Shimadzu model GC15 (Japan) fitted with a Shimadzu Chromatopac C-R4A chromatogram integrator. Sample injection was carried out using an OC-9 capillary on a column injector. A fused silica capillary column with chemically bonded phenylmethyl silicon liquid phase (5%) was used (DB-5, 0.25 mm i.d., J & W, San Marcos, CA, USA) for the GC analysis.

#### 2.1.6. High-Performance Liquid Chromatography (HPLC) Analysis

Aflatoxin B1 (C_17_H_12_O_6_) is a colorless to pale yellow crystal or white powder having a molecular weight of 312.27. HPLC (Shimadzu) with solvent modules (127 pumps) and a programmable UV detector. The isocratic mobile chromatography phase comprised H_2_O (80%) and MeOH (20%) (*v*/*v*). A guard column-protected 5 µm reverse-phase column (LiChrosphere^®^ C18; 250 mm × 4 mm non-polar) filtered the mobile phase and pumped it to waste (1 mL/min). A wavelength of 375 nm was adjusted, whereas the system had an injection loop of 10 µL. The pressures (50–350 bars) were frequently applied to push the liquid mobile phase through the column having a solid stationary phase. Freshly prepared aflatoxin samples (100 µL) were injected into the system. Before that, pumps were primed, and methanol was used for the first run to clean the injection loop and column from the impurities.

### 2.2. Preparation of Probiotic Inoculum 

A previously characterized probiotic strain (Pro 7, *Lactobacillus rhamnosus*) from camel milk (Accession No. MG890627) was used during this study [18]. Strain Pro7 was individually grown in standard (250 mL broth) De Man, Rogosa, and Sharp (MRS) culture medium (HiMedia) (37 °C for 72 h, under anaerobic conditions; growth was spectrophotometrically monitored at 600 nm). After incubation, the bacterial culture was centrifuged for 30 min at 5000× *g*. Then, a saline buffer was used to wash the cell pellet twice, followed by resuspension in phosphate buffer (50 mM, pH 6.8). Dilution and plating methods were adopted to estimate the final count (10^7^ CFU/mL) using MRS agar plates, and the suspension was kept at 4 °C until use.

### 2.3. Animals

Thirty adult male Wistar albino rats (180–200 g in weight and 7–8 weeks old) were obtained from the College of Pharmacy, Umm Al-Qura University, Makkah, Saudi Arabia. Sterile and polypropylene cages were used to house the rats in a normal 12 h light/dark cycle throughout the experimental duration (21 days). Before the initiation of experiments, the rats were kept in air-conditioned rooms (21–23 °C and 60–65% humidity) for two weeks and fed on a basic diet to ensure normal behavior and growth.

#### 2.3.1. Experimental Design

Thirty rats were randomly divided into six groups (five each). The control (Co) group was fed on a basal chow pellet diet and water. The milk (MK) group orally received 500 µL of milk for 21 days and was fed on a basal diet. The LAB group was orally given 500 µL of LAB suspension (10 mL/kg of body weight) for 21 days and fed on a basal diet [19]. AF group was orally administered with aflatoxin (0.5 mg/kg of body weight) for 21 days and fed on a basal diet [20]. The AF + LAB group was orally given aflatoxin (0.5 mg/kg) and LAB suspension (500 µL) for 21 days. The AF/LAB group was orally administered with aflatoxin (0.5 mg/kg) for 21 days, followed by an oral treatment of LAB suspension (500 µL) for 21 days.

#### 2.3.2. Biochemical Study

The rats were sacrificed after experiments (21 days), and blood samples were collected through cardiac puncture. The blood of each animal was split into two blood sampling tubes: (a) a plain tube for biochemical assays and (b) a tri-potassium ethylenediaminetetraacetic acid (K3-EDTA)-containing tube for hematological assays. Blood samples of the EDTA tube were examined for complete blood count (CBC) (hemoglobin content, red blood cells (RBCs), mean corpuscular hemoglobin (MCH), mean corpuscular volume (MCV), mean corpuscular hemoglobin concentration (MCHC), white blood cells (WBCs), platelet count, and neutrophil, monocyte, lymphocyte, basophil, and eosinophil counts). Blood was collected in a plain tube and centrifuged (3000 rpm) for 15 min for serum extraction. Serum aliquot was stored at −80 °C for further kidney function tests (creatinine, uric acid, and urea) and liver function tests (bilirubin, alanine aminotransferase (ALT), aspartate aminotransferase (AST), alkaline phosphatase (ALP), albumin, and total proteins). Rat-specific ELIZA kits (Bender MedSystems Gmbh, Wien, Austria) were used to determine inflammatory tissue biomarkers (lactate dehydrogenase (LDH) and creatinine kinase (CK)).

#### 2.3.3. Histological Study

The kidneys, liver, spleen, and testes were immediately isolated, rinsed in water to remove blood, and kept in formalin (10%) at room temperature for 24 h. The specimens were taken from the buffered formalin and processed through a graded series of xylene (Merck KGaA) and ethanol (Merck KGaA). Then, the specimens were paraffin-embedded, sectioned using a rotary microtome (5-micron thickness), slide-mounted, and stained with hematoxylin-eosin [21]. The slides were examined under a light microscope (Olympus) (Evident, Tokyo, Japan).

### 2.4. Statistical Analysis 

Data were expressed as mean +/− standard error. IBM SPSS Statistics software (version 23) was used for data analysis, whereas normal data distribution was evaluated through the Shapiro–Wilk test. Univariate analysis using OneWay ANOVA, followed by Duncan’s Multiple Range Test (DMRT), was utilized to calculate significance. The *p*-values of <0.05 were considered statistically significant. 

## 3. Results

AF (*p* < 0.050) and AF + LAB (*p* < 0.010) groups presented a significant decrease in RBC count compared to the control, whereas the RBC counts were significantly higher in the MK and AF/LAB groups than the AF group (*p* < 0.001). MCV remained significantly high in the AF/LAB group in comparison to the AF group (*p* < 0.050), whereas the platelet count significantly differentiated in all the studied groups (Table 1).

WBC counts remained significantly lower in control (*p* < 0.001), LAB (*p* < 0.010), and AF/LAB (*p* < 0.010) groups compared to the AF group, whereas lymphocyte counts were significantly higher in control (*p* < 0.010), MK (*p* < 0.050), LAB (*p* < 0.010), and AF + LAB (*p* < 0.001) groups in comparison to the AF group. The control, MK, and LAB groups presented significantly reduced neutrophil counts than the AF group (*p* < 0.001). Monocyte counts were significantly high in the AF (*p* < 0.001), AF + LAB (*p* < 0.050), and AF/LAB (*p* < 0.010) groups compared to the control group. Contrarily, monocyte counts remained significantly lower in the MK, LAB, AF + LAB, and AF/LAB groups than in the AF group (*p* < 0.001). The AF (*p* < 0.001), AF + LAB (*p* < 0.001), and AF/LAB (*p* < 0.010) groups demonstrated significantly decreased eosinophil counts compared to the control group. However, eosinophil counts were significantly high in the LAB group compared to the AF group (*p* < 0.001) (Table 2).

Significantly decreased levels of serum urea and creatinine were noted in the kidney sections of the control, MK, LAB, AF + LAB, and AF/LAB groups compared to those of the AF group (*p* < 0.001). Serum uric acid levels were significantly decreased in the AF (*p* < 0.001), AF + LAB (*p* < 0.001), and AF/LAB (*p* < 0.010) groups compared to the control group, whereas they significantly increased in the MK, LAB, AF + LAB, and AF/LAB groups in comparison to the AF group (*p* < 0.010) (Table 3).

The liver sections of the AF group presented a significant rise in serum AST, ALT, and ALP levels compared to the control, MK, LAB, AF + LAB, and AF/LAB (*p* < 0.001) groups. ALP serum levels were significantly higher in the AF + LAB and AF/LAB groups than in the control (*p* < 0.001) group. Serum total protein and albumin levels remained significantly high in the control, MK, LAB, AF + LAB, and AF/LAB groups compared to the AF group (*p* < 0.010), whereas serum total bilirubin levels significantly decreased in control (*p* < 0.001), MK (*p* < 0.010), LAB (*p* < 0.010), AF + LAB (*p* < 0.010), and AF/LAB (*p* < 0.010) groups in comparison to the AF group (Table 4).

Significantly lower LDH and CK serum levels were observed in control, MK, LAB, AF + LAB, and AF/LAB groups than in the AF group (*p* < 0.001). However, these levels were significantly higher in the AF + LAB (*p* < 0.001) and AF/LAB (*p* < 0.001) groups compared to the control group (Table 5). 

**Figure 1:**
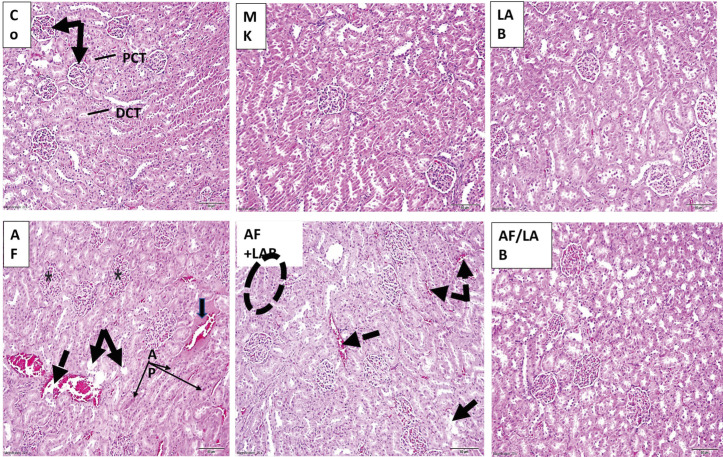
Photomicrographs of kidney sections from control, MK, and LAB groups depicting the normal histological structure of glomeruli (arrows), proximal convoluted tubules (PCTs), and distal convoluted tubules (DCTs). AF group presented glomeruli with dilated congested capillaries, expanded mesangial matrix, and loss of the subcapsular space (star). Vacuolation of renal tubules (arrows), hemorrhage (dotted arrow), sporadic coagulative necrosis with interstitial hemorrhage (thick arrow), tubular cells with highly eosinophilic cytoplasm, and pyknotic nuclei (apoptotic) (AP) are also shown in AF group. The kidney section of AF + LAB presented sporadically dilated intertubular congestion (dotted arrows), vacuolated tubular cells (arrow), and localized apoptosis (circle). AF/LAB group demonstrated structural improvement with minimal glomerular congested capillaries (H&E ×200).

Figure 1 depicts the effects of MK, LAB, AF, AF + LAB, and AF/LAB administrations on the kidney structure. The kidney histology of the control, MK, and LAB groups revealed the normal histological structure of glomeruli, proximal convoluted tubules (PCTs), and distal convoluted tubules (DCTs). Contrarily, glomeruli with dilated congested capillaries, an expanded mesangial matrix, and a loss of subcapsular space were noted in the AF-treated group. The renal tubules demonstrated vacuolation, sporadic coagulative necrosis with interstitial hemorrhage, and tubular cells with pyknotic nuclei and highly eosinophilic cytoplasm. The kidneys of the AF + LAB group presented sporadic dilation of intertubular congestion, tubular cell vacuolation, and localized apoptosis. However, a structural improvement was noted in the AF/LAB group, with minimal glomerular congested capillaries.

**Figure 2:**
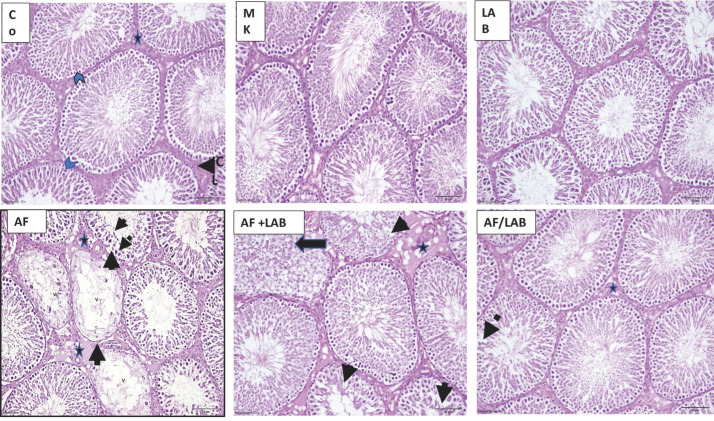
Photomicrographs of liver sections from control, MK, and LAB groups reveal the normal histological structure as well as hepatocytes radiating from the central vein (CV) separated by sinusoids (arrows). AF group demonstrated congested dilated portal vein (PV) with localized wall disruption (head arrow), periportal infiltration of polymorphonuclear lymphocytes (PNL) (arrow), dilated congested sinusoids (dashed arrows), apoptotic hepatocytes (circle), and vacuolated nuclei (square). The liver sections of the AF + LAB group revealed congested portal vein, PNL infiltration (dashed arrows), and apoptotic periportal hepatocytes (circle). Congested central vein (CV) and sinusoids (dashed arrows) and sporadic hepatocyte nuclei vacuolation were detected in AF/LAB group (arrows) (H&E ×200).

Normal histological structures were observed in the liver photomicrographs of the control, MK, and LAB groups. Contrarily, the livers of the AF-treated rats revealed congestion and dilation of the portal vein with localized wall disruption, dilated congested liver sinusoids, and periportal infiltration with polymorph nuclear leucocytes. Vacuolated nuclei and apoptosis were noted in hepatocytes as well. AF + LAB treatment resulted in a congested portal vein, apoptotic periportal hepatocytes, and polymorphonuclear lymphocyte (PNL) infiltration in the liver. Sporadic vacuolation of the hepatocyte nuclei and congested sinusoids and central veins were noted in the AF/LAB group (Figure 2).

**Figure 3:**
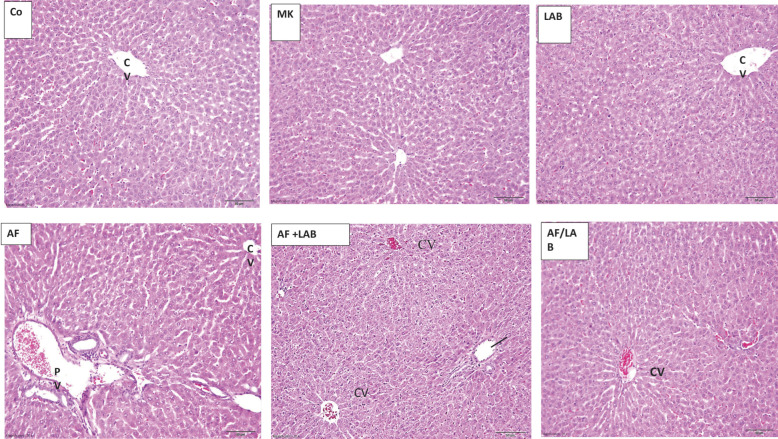
Photomicrographs of spleen sections of control, MK, and LAB groups show the normal histological structure of the central artery (CA), the red pulp (RP), and the white pulp (WP). The spleen sections of the AF group revealed lymphoid depletion in WP (thick arrow), localized hemorrhage (star), and hemosiderin pigment deposition (arrows). Scattered hemosiderin pigment (arrows) was noted in spleen sections of the AF + LAB group with hemorrhage in RP. There were only a few hemosiderin deposits in the AF/LAB group (H&E ×200).

The spleen photomicrographs of the control, MK, and LAB groups presented normal histological structures. The spleen sections of the AF-treated rats revealed a depletion of lymphoids in the white pulp, localized hemorrhage, and hemosiderin pigment deposition. The spleen sections of the AF + LAB group presented scattered hemosiderin pigment in hemorrhage areas of the red pulp; there were only a few hemosiderin deposits in the spleen sections of this group (Figure 3).

**Figure 4:**
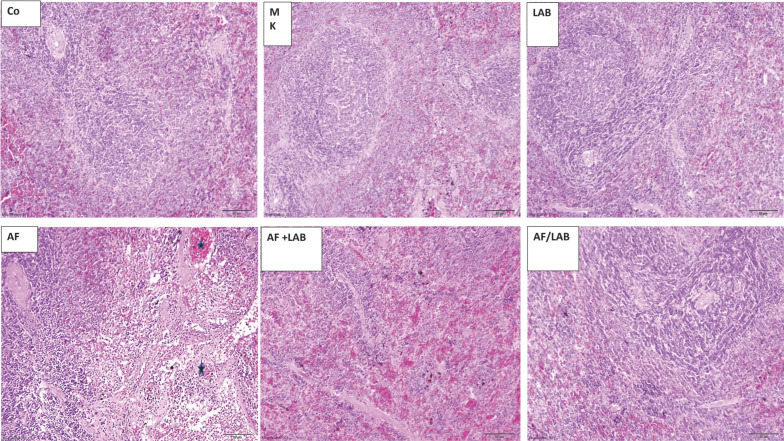
Photomicrographs of testes sections from control, MK, and LAB groups reveal the normal histological structure of seminiferous tubules (STs) (head arrows). The interstitial cells of Leydig (ICL) and interstitial space (star) were in the normal range. Testes sections of AF groups demonstrated vacuolated ST with completely lost spermatogenic cells (V), widened interstitial space (star), germinal epithelium detachment from the basement membrane (arrow), and reduced spermatogenic cell (dashed arrows) numbers in some tubules. An alteration in ST structure (thick arrow), vacuolation and widening of interstitial space (star), and localized vacuolation areas (arrows) were noted in the AF + LAB group. Regaining of the normal structure was noted in AF/LAB group except for sporadic vacuolation in tubules (dotted arrows) and interstitial space (star) (H&E ×200).

The photomicrographs of the testis sections from the control, MK, and LAB groups depicted the normal histological structure of seminiferous tubules with all stages of spermatogenic and Sertoli cells. The interstitial spaces normally have interstitial Leydig cells. The testes of the AF group demonstrated interstitial space widening, vacuolated ST with completely lost spermatogenic cells, and germinal epithelium detachment from the basement membrane. The spermatogenic cell number also decreased in some tubules. The normal structure of some of the ST was altered in the AF + LAB group, along with the vacuolation and widening of the interstitial space. Most tubules of the AF/LAB group regained their normal structure, except for the sporadic vacuolation of the interstitial space and tubules (Figure 4). 

## 4. Discussion

Long-term aflatoxin food contaminations are common in developing countries. Improper crop harvesting, handling, and storage facilitate fungal growth. Aflatoxins are well-known for their carcinogenic, hepatotoxic, and organ-specific toxicities. Aflatoxins and their metabolic byproducts are also associated with the malabsorption of nutrients and a weakened immune system, which can lead to dietary deficiencies (marasmus and kwashiorkor), stunted growth, and poor immunological function [22]. Probiotics systematically benefit the immune system, metabolism, and health of the host [23]. This study investigated the efficacy of oral LAB administration in alleviating AFB1 toxicity in different rat organs (spleen, kidney, testes, and liver).

AF-exposed rats exhibited significantly decreased RBC, lymphocyte, and eosinophil counts. Contrarily, a significant increase in WBC, neutrophil, and monocyte counts was noted compared to the control. LAB administration with/after AF (AF + LAB and AF/LAB groups) improved the AF-caused hematological alterations. Ramamurthy and Rajakumar [24] and Khaled and Thalij [25] have reported a significant decrease in hemoglobin content and RBCs and a significant rise in WBC count in rats fed an AFB1-treated diet in comparison to the control group. An RBC decline in AF-treated rats might be attributed to reduced erythropoietin hormone activity, which is secreted by the liver and kidneys. The decrease in hemoglobin content could be due to reduced erythrocyte volume, lower erythropoietin formation, and defective heme-biosynthesis in the bone marrow [25]. AFB1 particularly suppresses the T cells of the cellular immune system through macrophage phagocytosis, neutrophil activity, and a reduction in complement synthesis in the liver [26]. Mycotoxin interferes with lymphocyte function and receptors to induce cytotoxicity [27]. A WBC (mainly neutrophils) increase reflects the inflammatory response of these cells [25]. Yeasts and bacteria can neutralize the mycotoxins in the body by breaking, converting, and rearranging them into inactive forms or harmless metabolites [10,19]. LAB restrict mycotoxin absorption in the colon to facilitate their release with feces [28]. LAB peptidoglycans might bind with mutagenic mycotoxins to minimize their bioavailability and stability and further promote macrophage-based anti-inflammatory cytokine secretion [29]. Higher LAB concentrations could enhance native and memory T-lymphocyte proliferation and activation [30]. Carboxylic acids, organic acids, phenolic acids, sporulation-preventing chemicals, and cyclic dipeptides are the main metabolites of LAB, which reduce mycotoxin production to serve as antifungal agents [31].

During this study, AF administration significantly impaired kidney functions by enhancing creatinine and urea levels and decreasing uric acid levels in the serum. *Lactobacillus casei* Shirota (LcS) supplement has been reported to reduce serum urea levels in AF-administered rats [28,32]. The rise in creatinine and uric acid serum levels indicated kidney dysfunction and protein catabolism [19]. The results indicated the stressful impacts of AFs on renal tissues, which is in line with previous reports of aflatoxicosis [33,34]. AF administration could cause renal injury and cell necrosis, as depicted by the enhanced serum levels of uric acid, creatinine, and urea [35,36]. The results demonstrated that LAB administrations (AF + LAB and AF/LAB) ameliorated kidney functions. Hathout et al. [19] have also revealed that *Lact. Reuteri* and *Lact. Casei* treatments successfully prevented AF-based kidney injury, which was indicated by significantly improved creatinine and plasma urea levels.

The kidney histology results of this study were in line with the biochemical data and AF-based renal damage. A histological examination of the kidney revealed dilated glomeruli with congested capillaries and an expanded mesangial matrix with the loss of subcapsular space in the AF group. Renal tubules were characterized by vacuolation, sporadic coagulative necrosis with interstitial hemorrhage, pyknotic nuclei, and highly eosinophilic cytoplasm in some tubular cells. AF-administration-based renal tubule necrosis, expanded pale vacuolated cytoplasm, and granular degeneration with proximal tubules’ epithelial cell swelling has been documented by various researchers [37,38,39]. The kidney sections of the AF + LAB group presented a sporadic dilation of intertubular congestion, tubular cell vacuolation, and localized apoptosis. A significant structural improvement was noted in the AF/LAB group, with minimal glomerular congested capillaries. The results indicated better LAB therapeutic efficacy than protective effects on the kidney. Śliżewska et al. [40] reported the absence of histological changes in the kidneys of chickens fed probiotic-supplemented diets containing 1 mg AFB1/kg, whereas histological alterations of lower intensity were observed when chickens were fed a probiotic-supplemented diet containing 5 mg AFB1/kg. LAB and probiotic yeast are known to efficiently bind AFB1, followed by its conversion to a nontoxic molecule [41,42]. The histological abnormalities were overall lesser than the birds fed the contaminated but non-supplemented diet. Probiotic supplements might have restricted the toxin’s reach to the kidneys and liver.

The data of this study reveal significantly enhanced serum levels of AST, ALT, ALP, and total bilirubin, whereas total proteins and albumin levels considerably decreased in the AF group compared to controls. Higher AST, ALT, and ALP levels might indicate liver hypofunction and degeneration [19,32]. However, these conditions were significantly improved in the AF + LAB and AF/LAB groups in comparison to the AF group. Nikbakht Nasrabadi et al. [32] reported significantly reduced AST and ALT levels in probiotics (LcS)-treated rats. Hathout et al. [19] reported the successful prevention of AFB-based hepatic injury by using two probiotics (*Lact. Reuteri* and *Lact. Casei*), which significantly improved AST and ALT plasma levels. The liver histology of the AF group depicted congestion and dilation of the portal vein with localized wall disruption, dilation and congestion of liver sinusoids, PNL infiltration of the periportal region, vacuolated hepatocyte nuclei, and apoptosis. Multiple studies have reported necrosis; dysplastic, prominent lesions; enlarged hepatocytes; and severe hydropic degeneration in response to AFB1 administration [37,43,44]. The liver metabolization of AFB1 produces a highly reactive epoxide (AFB1-8, 9-epoxide) that is injurious to hepatic cells [45]. Meanwhile, the liver sections of the AF + LAB group showed congestion of the portal vein, PNL infiltration, and apoptotic periportal hepatocytes. The AF/LAB group demonstrated the congestion of sinusoids and central veins with sporadic hepatocyte nuclei vacuolation. Hathout et al. [19] also revealed LAB-treatment-based prevention of AF hepatic injury, which is evident in the hepatic histological picture. LAB bind AFs in the gastrointestinal tract to reduce their bioavailability [46]. Kodali and Sen [47] have suggested another probiotic-bacteria-based prevention mechanism, which involves the extracellular synthesis of polysaccharides to exert therapeutic, physiological, antioxidant, and free-radical-scavenging impacts. This is in line with the findings of Hathout et al. [19], who reported decreased accumulation risk of reactive oxygen species along with hydrogen peroxide and superoxide anion degradation in response to LAB treatment [48]. Moreover, cell lysates and intact cells of some probiotic strains (*L. fermentum E18* and *Lactobacillus fermentum E3*) possess antioxidant characteristics to counter oxidative stress [49]. The cytoplasmic fractions of *Lactobacillus acidophilus*, *Lactobacillus delbreukii* ssp. *lactis*, *L. casei*, and *L. delbreukii* ssp. *bulgaricus* are also known to exhibit high antioxidant activities [50].

The spleen, the largest peripheral lymphoid tissue, is associated with the total immune function of the body. During this study, rat spleen sections of the AF group presented lymphoid depletion in the white pulp, hemosiderin pigment deposition, and localized hemorrhages. AFB1-associated rat spleen damage is characterized by lower spleen mononuclear cell (SMC) proliferation [51], biomolecular oxidative damage, a mutation of splenic lymphocytes [52], and decreased CD8+ T and CD4+ T cell numbers [53]. Omar [54] has reported the vacuolar degeneration of spleen cells after AFB administration in rats. The reduced inflammatory response through AFB1-based Kupffer cell suppression in the liver is similar to the suppression of spleen histopathology changes and macrophage functions [55]. Dietary aflatoxin linked to broilers’ immunosuppression affects the thymus, spleen, and bursa of Fabricius [56]. This study revealed scattered hemosiderin pigments in spleen sections within hemorrhage areas in the red pulp of the AF + LAB group, whereas a few hemosiderin deposits were observed in the AF/LAB group. These partial improvements could be associated with the LAB-based reduction in AFB1 toxicity. The testes of the AF group demonstrated vacuolated STs with completely lost spermatogenic cells, widened interstitial space, germinal epithelium detachment from the basement membrane, and lower spermatogenic cell numbers. Kudayer et al. [57] revealed that AFB1 administration to rats for seven days resulted in excessively vacuolated testicular cells with suppressed spermatogenesis. These results are in line with previous studies, which have established AF’s toxicity to the reproductive system [58,59]. AFB1 induces the necrosis and degeneration of the testes’ seminiferous tubule epithelium lining [59]. Deabes et al. [60] noted that oral AF administration adversely affected the male reproductive system of mice. Different studies have reported similar findings in various animals and established AFs as spermatogenesis-disrupting reproductive toxicants, which lead to defective spermatozoa production [61,62,63,64]. Meanwhile, the abnormal structure of some STs with interstitial space vacuolation and widening was noted in the AF + LAB group during this study. The examination of AF/LAB revealed a regaining of the normal tubular structure except for sporadic vacuolation in the interstitial space and tubules. Similarly, an investigation has reported that Lactobacillus rhamnosus GG ATCC53013 (LGG) pretreatment could significantly mitigate the mycotoxin-induced alterations in mice reproductive parameters such as enhanced sperm motility, increased sperm number, and reduced sperm abnormalities [60]. During this study, the serum levels of tissue destruction markers (CK and LDH) significantly increased in the AF, AF + LAB, and AF/LAB groups as compared to control, whereas they were significantly lower in the AF + LAB and AF/LAB groups in comparison to the AF group. LAB might act as anti-inflammatory agents to reduce AFB1-associated oxidative stress [10]. Probiotics could stimulate humoral immune cells, T cell subsets, macrophages, and epithelial-associated dendritic cells to enhance the capability of anti-inflammatory cytokines [65]. LAB might improve the capacity of leukocytes’ phagocytic receptor (complement receptor 3 (CR3)) to bluff respiratory bursts [66].

## 5. Conclusions 

This study concludes that oral *Lactobacillus rhamnosus* (LAB) administration could effectively counter AFB1-induced disruptions in different organs (kidneys, liver, spleen, and testes) of male rats. Significant improvements in blood parameters altered by the effect of aflatoxin were observed in rats administered with *L. rhamnosus*. Moreover, kidney functions were significantly ameliorated after aflatoxin-exposed rats were administered with the bacteria. Similar significant improvements in the functions of the liver, spleen, and testes were noted when *L. rhamnosus* was given to rats exposed to AF. This may suggest that the therapeutic impacts of *L. rhamnosus* strain used in this study were more pronounced than the protective effects. The protective and therapeutic efficacy of *L. rhamnosus* against aflatoxigenic fungi can be attributed to their bioactive metabolites, AF’s binding capability, and antioxidant and anti-inflammatory properties.

**Practical Implications:** The *L. rhamnosus* strain explored in this study has been previously exhibiting anti-foodborne pathogen activities, as well as potentially improving the immune response of the intestinal mucosa. Together with its therapeutic capabilities, and aflatoxins-induced damage on various organs, it can be employed for the competitive biological exclusion of foodborne pathogens to avoid fungal toxins in consumable products.

## Figures and Tables

**Table 1:** Effects of different oral administrations on rats’ RBCs, hemoglobin, MCV, MCH, MCHC, and platelets.

Variables	Control	MK	LAB	AF	AF + LAB	AF/LAB
**RBCs** (×10^6^/UL)	9.12 ± 0.19	9.33 †** ± 0.09	9.02 ± 0.12	8.44 ± 0.21 *	8.32 ‡** ± 0.05	9.37 †*** ± 0.13
**Hemoglobin** (g/dL)	15.64 ± 0.30	15.84 ± 0.33	15.72 ± 0.34	16.36 ± 0.38	15.50 ± 0.18	15.96 ± 0.25
**Hematocrit** (%)	47.30 ± 1.12	48.34 ± 1.10	47.34 ± 0.82	49.42 ± 1.13	45.70 ± 0.41	48.94 ± 0.75
**MCV** (fL)	51.86 ± 0.22	51.84 ± 1.00	53.10 ± 1.32	51.26 ± 0.84	52.26 ± 0.50	54.94 ± 0.39 †*
**MCH** (pg/dL)	17.14 ± 0.11	16.26 ± 0.61	17.38 ± 0.39	17.10 ± 0.08	17.96 ± 0.42	17.02 ± 0.12
**MCHC** (g/dL)	33.06 ± 0.23	32.80 ± 0.17	33.20 ± 0.13	33.10 ± 0.31	32.42 ± 0.24	33.90 ± 0.09
**Platelets** (×10^3^/UL)	1064.40 ± 10.73	1073.80 ± 15.02	1085.80 ± 23.26	1044.80 ± 11.54	1099.20 ± 12.86	1039.20 ± 9.67

MK: milk, LAB: lactic acid bacteria, AF: aflatoxin, RBCs: red blood cells; MCV: mean corpuscular volume; MCH: mean corpuscular hemoglobin; MCHC: mean corpuscular hemoglobin concentration. ‡: significance versus control; †: significance versus aflatoxin. *: *p* < 0.050; **: *p* < 0.010; ***: *p <* 0.001.

**Table 2:** Effects of different oral administrations on rats’ WBC, lymphocyte, neutrophil, monocyte, eosinophil, and basophil counts.

Variables	Control	MK	LAB	AF	AF + LAB	AF/LAB
**WBCs** (×10^3^/UL)	15.01 ± 0.61	18.61 ± 1.87	16.47 †** ± 0.70	22.27 ‡*** ± 1.28	19.31 ± 0.75	16.35 †** ± 0.86
**Lymphocytic count** (×10^3^/UL)	12.25 ± 0.37	12.39 ± 0.37 †**	12.10 ± 0.39 †*	9.20 ‡** ± 0.28	14.09 †*** ± 0.99	11.51 ± 0.62
**Neutrophil count** (×10^3^/UL)	2.53 ± 0.17	2.44 †*** ± 0.19	2.49 †*** ± 0.08	4.13 ‡*** ± 0.25	3.08 ± 0.32	3.15 ± 0.35
**Monocyte count** (×10^3^/UL)	1.53 ± 0.06	1.41 †*** ± 0.09	1.57 †*** ± 0.15	4.58 ± 0.33 ‡***	2.41 ‡*/†*** ± 0.18	2.50 ‡**/†*** ± 0.12
**Eosinophil count** (×10^3^/UL)	0.77 ± 0.06	0.52 ± 0.07	0.72 †*** ± 0.09	0.28 ‡*** ± 0.02	0.27 ‡*** ± 0.03	0.46 ‡** ± 0.02
**Basophil count** (×10^3^/UL)	0.04 ± 0.004	0.05 ± 0.005	0.04 ± 0.004	0.04 ± 0.005	0.05 ± 0.002	0.04 ± 0.007

MK: milk, LAB: lactic acid bacteria, AF: aflatoxin, ‡: significance versus control; †: significance versus aflatoxin. *: *p* < 0.050; **: *p* < 0.010; ***: *p <* 0.001.

**Table 3:** Effects of oral administration on rats’ kidney functions.

Variables	Control	MK	LAB	AF	AF + LAB	AF/LAB
**Urea** (mg/dL)	18.08 ± 1.27	17.58 †*** ± 1.09	17.98 †*** ± 0.82	23.74 ‡*** ± 0.42	17.58 †*** ± 0.47	16.60 †*** ± 0.45
**Creatinine** (mg/dL)	0.20 ± 0.01	0.20 †*** ± 0.01	0.20 †*** ± 0.01	0.33 ‡*** ± 0.01	0.22 †*** ± 0.01	0.21 †*** ± 0.01
**Uric acid** (mg/dL)	1.56 ± 0.07	1.52 †*** ± 0.11	1.66 †*** ± 0.02	0.76 ‡*** ± 0.06	1.06 †/‡*** ± 0.04 †/‡***	1.26 ‡**/†*** ± 0.09

MK: milk, LAB: lactic acid bacteria, AF: aflatoxin, ‡: significance versus control; †: significance versus aflatoxin. **: *p* < 0.010; ***: *p <* 0.001.

**Table 4:** Effects of oral administration on rats’ liver parameters.

Variables	Control	MK	LAB	AF	AF + LAB	AF/LAB
**AST** (U/L)	111.74 ± 3.40	110.86 †*** ± 2.72	111.06 †*** ± 0.97	152.14 ‡*** ± 2.79	109.52 †*** ± 1.19	106.76 †*** ± 1.31
**ALT** (U/L)	78.12 ± 2.30	81.68 †*** ± 1.67	77.08 †*** ± 3.15	122.84 ‡*** ± 3.39	80.58 †*** ± 4.94	75.32 †*** ± 1.03
**ALP** (U/L)	75.40 ± 3.52	81.80 †*** ± 4.68	69.80 †*** ± 5.54	186.20 ‡*** ± 4.18	101.80 †/‡*** ± 3.43	100.60 †/‡*** ± 2
**Total proteins** (mg/dL)	5.49 ± 0.35	5.38 †*** ± 0.22	5.13 †*** ± 0.39	3.84 ‡*** ± 0.23	5.43 †*** ± 0.15	5.44 †*** ± 0.32
**Albumin** (g/dL)	3.55 ± 0.17	3.58 †*** ± 0.10	3.20 †*** ± 0.23	1.43 ‡*** ± 0.11	3.20 †*** ± 0.06	3.45 †*** ± 0.14
**Bilirubin** (mg/dL)	0.03 ± 0.01	0.03 †** ± 0.01	0.03 †** ± 0.01	0.05 ‡*** ± 0.002	0.03 †** ± 0.002	0.03 †** ± 0.002

MK: milk, LAB: lactic acid bacteria, AF: aflatoxin, AST: aspartate aminotransferase, ALT: alanine aminotransferase, ALP: alkaline phosphatase. ‡: significance versus control; †: significance versus aflatoxin. **: *p* < 0.010; ***: *p <* 0.001.

**Table 5:** Effects of oral administrations on rats’ tissue destruction markers.

Variables	Control	MK	LAB	AF	AF + LAB	AF/LAB
**LDH** (mg/dL)	226.60 ± 3.31	238.40 †*** ± 7.39	244.00 †*** ± 8.14	463.80 ‡*** ± 10.77	336.80 †/‡*** ± 5.29	336.80 †/‡*** ± 6.92
**CK** (IU/L)	211.40 ± 6.13	202.00 †*** ± 6.57	199.80 †*** ± 3.01	385.40 ‡*** ± 3.53	285.80 †/‡*** ± 4.01	232.80 ‡**/†*** ± 3.89

MK: milk, LAB: lactic acid bacteria, AF: aflatoxin, LDH: lactate dehydrogenase; CK: creatinine kinase. ‡: significance versus control; †: significance versus aflatoxin. **: *p* < 0.010; ***: *p <* 0.001.

## Data Availability

All datasets generated and gathered during this research are included in the manuscript.

## References

[1] Shan Y. (2019). The toxic effects of aflatoxin B1: An update. Aflatoxin B1 Occurrence, Detection and Toxicological Effects.

[2] Pickova D., Ostry V., Toman J., Malir F. (2021). Aflatoxins: History, Significant Milestones, Recent Data on Their Toxicity and Ways to Mitigation. Toxins.

[3] Rushing B.R., Selim M.I. (2019). Aflatoxin B1: A review on metabolism, toxicity, occurrence in food, occupational exposure, and detoxification methods. Food Chem. Toxicol..

[4] Gao Y., Ye Q., Bao X., Huang X., Wang J., Zheng N. (2020). Transcriptomic and proteomic profiling reveals the intestinal immunotoxicity induced by aflatoxin M1 and ochratoxin A. Toxicon.

[5] Misra S., Pandey P., Mishra H.N. (2021). Novel approaches for co-encapsulation of probiotic bacteria with bioactive compounds, their health benefits and functional food product development: A review. Trends Food Sci. Technol..

[6] Wu J., Zhang Y., Ye L., Wang C. (2020). T1he anti-cancer effects and mechanisms of lactic acid bacteria exopolysaccharides in vitro: A review. Carbohydr. Polym..

[7] Aween M.M., Hassan Z., Muhialdin B.J., Noor H.M., Eljamel Y.A. (2012). Evaluation on Antibacterial Activity of Lactobacillus acidophilus Strains Isolated from Honey. Am. J. Appl. Sci..

[8] Rämö S., Kahala M., Joutsjoki V. (2022). Aflatoxin B1 Binding by Lactic Acid Bacteria in Protein-Rich Plant Material Fermentation. Appl. Sci..

[9] Jebali R., Abbès S., Ben Salah-Abbès J., Ben Younes R., Haous Z., Oueslati R. (2015). Ability of *Lactobacillus plantarum* MON03 to mitigate aflatoxins (B_1_and M_1_) immunotoxicities in mice. J. Immunotoxicol..

[10] Abbès S., Ben Salah-Abbès J., Jebali R., Ben Younes R., Oueslati R. (2016). Interaction of aflatoxin B_1_and fumonisin B_1_in mice causes immunotoxicity and oxidative stress: Possible protective role using lactic acid bacteria. J. Immunotoxicol..

[11] Sundaram B., Krishnamurthy R., Subramanian S. (1988). Aflatoxin-producing fungi in stored paddy. Proc. Plant Sci..

[12] Fakruddin, Chowdhury A., Hossain N., Ahmed M.M. (2015). Characterization of aflatoxin producing Aspergillus flavus from food and feed samples. Springerplus.

[13] Sreekanth D., Sushim G., Syed A., Khan B., Ahmad A. (2011). Molecular and Morphological Characterization of a Taxol-Producing Endophytic Fungus, *Gliocladium* sp., from *Taxus baccata*. Mycobiology.

[14] Nielsen K.F., Smedsgaard J. (2003). Fungal metabolite screening: Database of 474 mycotoxins and fungal metabolites for dereplication by standardised liquid chromatography–UV–mass spectrometry methodology. J. Chromatogr. A.

[15] Schmitz N., Laverty S., Kraus V.B., Aigner T. (2010). Basic methods in histopathology of joint tissues. Osteoarthr. Cartil..

[16] Bertrand S., Schumpp O., Bohni N., Bujard A., Azzollini A., Monod M., Gindro K., Wolfender J.-L. (2013). Detection of metabolite induction in fungal co-cultures on solid media by high-throughput differential ultra-high pressure liquid chromatography–time-of-flight mass spectrometry fingerprinting. J. Chromatogr. A.

[17] Saito M., Machida S. (1999). A rapid identification method for aflatoxin-producing strains of Aspergillus flavus and A. parasiticus by ammonia vapor. Mycoscience.

[18] Elbanna K., El Hadad S., Assaeedi A., Aldahlawi A., Khider M., Alhebshi A. (2018). In vitro and in vivo evidences for innate immune stimulators lactic acid bacterial starters isolated from fermented camel dairy products. Sci. Rep..

[19] Hathout A.S., Mohamed S.R., El-Nekeety A.A., Hassan N.S., Aly S.E., Abdel-Wahhab M.A. (2011). Ability of Lactobacillus casei and Lactobacillus reuteri to protect against oxidative stress in rats fed aflatoxins-contaminated diet. Toxicon.

[20] Yilmaz S., Kaya E., Karaca A., Karatas O. (2018). Aflatoxin B1 induced renal and cardiac damage in rats: Protective effect of lycopene. Res. Veter. Sci..

[21] Corrin B. (1981). Carleton’s histological technique. J. Clin. Pathol..

[22] Bbosa G.S., Kitya D., Lubega A., Ogwal-Okeng J., Anokbonggo W.W., Kyegombe D.B. (2013). Review of the biological and health effects of aflatoxins on body organs and body systems. Aflatoxins-Recent Adv. Future Prospect..

[23] Pandey K.R., Naik S.R., Vakil B.V. (2015). Probiotics, prebiotics and synbiotics—A review. J. Food Sci. Technol..

[24] Ramamurthy V., Rajakumar R. (2016). Studies on Ethanolic Leaf Extract of Phyllanthus Niruri and Its EFFECT on Aflatoxin Intoxicated Male Albino Rats. Int. J. Zool. Appl. Biosci..

[25] Khaled M.Q., Thalij K.M. (2021). Effect of Aflatoxin B1 Contaminated Corn and Their Products on Some Physiology Parameters in Laboratory Rats. IOP Conf. Ser. Earth Environ. Sci..

[26] Perdigón G., Fuller R., Raya R. (2001). Lactic acid bacteria and their effect on the immune system. Curr. Issues Intest. Microbiol..

[27] Tran V.N., Viktorová J., Ruml T. (2020). Mycotoxins: Biotransformation and Bioavailability Assessment Using Caco-2 Cell Monolayer. Toxins.

[28] Adilah Z.N., Liew W.-P.-P., Redzwan S.M., Amin I. (2018). Effect of High Protein Diet and Probiotic Lactobacillus casei Shirota Supplementation in Aflatoxin B1-Induced Rats. BioMed. Res. Int..

[29] Ghofrani Tabari D., Kermanshahi H., Golian A., Majidzadeh Heravi R. (2018). In vitro binding potentials of bentonite, yeast cell wall and lactic acid bacteria for aflatoxin B1 and ochratoxin A. Iran. J. Toxicol..

[30] Wells J.M. (2011). Immunomodulatory mechanisms of Lactobacilli. Microb. Cell Fact..

[31] Sadiq F.A., Yan B., Tian F., Zhao J., Zhang H., Chen W. (2019). Lactic Acid Bacteria as Antifungal and Anti-Mycotoxigenic Agents: A Comprehensive Review. Compr. Rev. Food Sci. Food Saf..

[32] Nasrabadi E.N., Jamaluddin R., Mutalib M.A., Khaza’Ai H., Khalesi S., Redzwan S.M. (2013). Reduction of aflatoxin level in aflatoxin-induced rats by the activity of probiotic *Lactobacillus casei* strain Shirota. J. Appl. Microbiol..

[33] Sherif S.O., Salama E.E., Abdel-Wahhab M.A. (2009). Mycotoxins and child health: The need for health risk assessment. Int. J. Hyg. Environ. Health.

[34] Abdel-Wahhab M., Kholif A. (2010). Mycotoxins in animal feeds and prevention strategies: A review. Asian J. Anim. Sci..

[35] Wang L. (2017). The significance of Cys-C UREA and Scr tests in early renal damage assessment of acute glomerulonephritis. Labeled Immunoass. Clin. Med..

[36] Solbu M.D., Norvik J.V., Storhaug H.-M., Eriksen B.O., Melsom T., Eggen A.E., Zykova S.N., Kronborg J.B., Jenssen T.G. (2016). The Association between Adiponectin, Serum Uric Acid and Urinary Markers of Renal Damage in the General Population: Cross-Sectional Data from the Tromsø Study. Kidney Blood Press. Res..

[37] Olonisakin O., Ogidi C., Jeff-Agboola Y., Akinyele B. (2019). Histopathological studies on kidney and liver of albino rat infected with toxigenic Aspergillus flavus after treatment with isolated Lactobacillus species from Kunu. Afr. J. Clin. Exp. Microbiol..

[38] Abdel-Wahhab M., Nada S., Khalil F. (2002). Physiological and toxicological responses in rats fed aflatoxin-contaminated diet with or without sorbent materials. Anim. Feed. Sci. Technol..

[39] El-Mahalaway A.M. (2015). Protective effect of curcumin against experimentally induced aflatoxicosis on the renal cortex of adult male albino rats: A histological and immunohisochemical study. Int. J. Clin. Exp. Pathol..

[40] Śliżewska K., Cukrowska B., Smulikowska S., Cielecka-Kuszyk J. (2019). The Effect of Probiotic Supplementation on Performance and the Histopathological Changes in Liver and Kidneys in Broiler Chickens Fed Diets with Aflatoxin B1. Toxins.

[41] Awad W., Ghareeb K., Abdel-Raheem S., Böhm J. (2009). Effects of dietary inclusion of probiotic and synbiotic on growth performance, organ weights, and intestinal histomorphology of broiler chickens. Poult. Sci..

[42] Liżewska K., Smulikowska S. (2011). Detoxification of aflatoxin B. J. Anim. Feed. Sci..

[43] Yener Z., Celik I., Ilhan F., Bal R. (2009). Effects of Urtica dioica L. seed on lipid peroxidation, antioxidants and liver pathology in aflatoxin-induced tissue injury in rats. Food Chem. Toxicol..

[44] Gelderblom W., Marasas W., Lebepe-Mazur S., Swanevelder S., Vessey C., Hall P.D.L.M. (2002). Interaction of fumonisin B1 and aflatoxin B1 in a short-term carcinogenesis model in rat liver. Toxicology.

[45] Rotimi O.A., Rotimi S.O., Duru C.U., Ebebeinwe O.J., Abiodun A.O., Oyeniyi B.O., Faduyile F.A. (2017). Acute aflatoxin B1—Induced hepatotoxicity alters gene expression and disrupts lipid and lipoprotein metabolism in rats. Toxicol. Rep..

[46] Salminen S., Nybom S., Meriluoto J., Collado M.C., Vesterlund S., El-Nezami H. (2010). Interaction of probiotics and pathogens—Benefits to human health?. Curr. Opin. Biotechnol..

[47] Kodali V.P., Sen R. (2008). Antioxidant and free radical scavenging activities of an exopolysaccharide from a probiotic bacterium. Biotechnol. J..

[48] Liu C.-F., Pan T.-M. (2010). In vitro effects of lactic acid bacteria on cancer cell viability and antioxidant activity. J. Food Drug Anal..

[49] Kullisaar T., Zilmer M., Mikelsaar M., Vihalemm T., Annuk H., Kairane C., Kilk A. (2002). Two antioxidative lactobacilli strains as promising probiotics. Int. J. Food Microbiol..

[50] Saide J., Gilliland S. (2005). Antioxidative Activity of Lactobacilli Measured by Oxygen Radical Absorbance Capacity. J. Dairy Sci..

[51] Mary V.S., Theumer M.G., Arias S.L., Rubinstein H.R. (2012). Reactive oxygen species sources and biomolecular oxidative damage induced by aflatoxin B1 and fumonisin B1 in rat spleen mononuclear cells. Toxicology.

[52] Morris S.M., Aidoo A., Chen J.J., Chou M.W., Casciano D.A. (1999). Aflatoxin B1-induced Hprt mutations in splenic lymphocytes of Fischer 344 rats.: Results of an intermittent feeding trial. Mutat. Res. Fundam. Mol. Mech. Mutagen..

[53] Sabourin P.J., Price J.A., Casbohm S.L., Perry M.R., Tuttle R.S., Rogers J.V., Rowell K.S., Estep J.E., Sabourin C.L. (2006). Evaluation of Acute Immunotoxicity of Aerosolized Aflatoxin B_1_in Female C57BL/6N Mice. J. Immunotoxicol..

[54] Omar N. (2012). Effect of some aflatoxins on a lymphatic organ (spleen) of male albino rats (histopathological study). Egypt. J. Hosp. Med..

[55] Hinton D.M., Myers M.J., Raybourne R.A., Francke-Carroll S., Sotomayor R.E., Shaddock J., Warbritton A., Chou M.W. (2003). Immunotoxicity of Aflatoxin B1 in Rats: Effects on Lymphocytes and the Inflammatory Response in a Chronic Intermittent Dosing Study. Toxicol. Sci..

[56] Ortatatli M., Oğuz H., Hatipoğlu F., Karaman M. (2005). Evaluation of pathological changes in broilers during chronic aflatoxin (50 and 100 ppb) and clinoptilolite exposure. Res. Veter. Sci..

[57] Kudayer A.M., Alsandaqchi A.T., Saleh F.M., Alwan N.A. (2019). Toxic Effect of Aflatoxin B1 on Heart, Lung, and Testis of Male Albino Rats: Histopathology Study. IOP Conf Ser. Mater. Sci. Eng..

[58] Nair A., Verma R. (2000). Effect of aflatoxin on histoarchitecture of testis of male mouse and its amelioration by vitamin E. Indian J. Toxicol..

[59] Murad A.F., Ahmed S., Abead S. (2015). Toxicity Effect of Aflatoxin B1 on Reproductive System of Albino Male Rats. Pak. J. Biol. Sci..

[60] Deabes M.M., Darwish H.R., Abdel-Aziz K.B., Farag I.M., Nada S.A., Tawfek N.S. (2012). Protective effects of Lactobacillus rhamnosus GG on Aflatox-ins-induced toxicities in male Albino Mice. J. Environ. Anal. Toxicol..

[61] Faridha A.F.K., Akbarsha M.A. (2006). Duration-dependent histopathological and histometric changes in the testis of aflatoxin B1-treated mice. J. Endocrinol. Reprod..

[62] Faridha A., Faisal K., Akbarsha M.A. (2007). Aflatoxin treatment brings about generation of multinucleate giant spermatids (symplasts) through opening of cytoplasmic bridges: Light and transmission electron microscopic study in Swiss mouse. Reprod. Toxicol..

[63] Agnes V., Akbarsha M. (2001). Pale vacuolated epithelial cells in epididymis of aflatoxin-treated mice. Reproduction.

[64] Agnes V., Akbarsha M. (2003). Spermatotoxic effect of aflatoxin B1 in the albino mouse. Food Chem. Toxicol..

[65] Braat H., Brande J.V.D., van Tol E., Hommes D., Peppelenbosch M., van Deventer S. (2004). Lactobacillus rhamnosus induces peripheral hyporesponsiveness in stimulated CD4+ T cells via modulation of dendritic cell function. Am. J. Clin. Nutr..

[66] Bravo M., Combes T., Martinez F.O., Cerrato R., Rey J., Garcia-Jimenez W., Fernandez-Llario P., Risco D., Gutierrez-Merino J. (2019). Lactobacilli Isolated from Wild Boar (*Sus scrofa*) Antagonize *Mycobacterium bovis* Bacille Calmette-Guerin (BCG) in a Species-Dependent Manner. Front. Microbiol..

